# A molecular assessment of *Ostertagia leptospicularis* and *Spiculopteragia asymmetrica* among wild fallow deer in Northern Ireland and implications for false detection of livestock-associated species

**DOI:** 10.1186/s13071-024-06147-2

**Published:** 2024-03-18

**Authors:** Maggie Lyons, Tony L. Brown, Angela Lahuerta-Marin, Eric. R. Morgan, Paul M. Airs

**Affiliations:** 1 School of Biological Sciences, Queen’s University Belfast, 19 Chlorine Gardens, Belfast, BT9 5DL UK; 2 Agri-Food and Biosciences Institute Northern Ireland, 12 Stoney Road, Belfast, Co Antrim, BT4 3SD UK

**Keywords:** Molecular diagnostics, PCR, Beta-tubulin, Benzimidazole resistance, Gastrointestinal nematodes

## Abstract

**Background:**

Wild deer populations utilizing livestock grazing areas risk cross-species transmission of gastrointestinal nematode parasites (GINs), including GINs with anthelmintic resistance (AR) traits. Wild deer have been shown to carry problematic GIN species such as *Haemonchus contortus* and *Trichostrongylus* species in the UK, but the presence of livestock GINs in Northern Ireland deer populations is unknown. Also, is it not known whether AR traits exist among GINs of deer such as *Ostertagia leptospicularis* and *Spiculopteragia asymmetrica* in pastureland where anthelmintics are heavily used.

**Methods:**

Adult-stage GIN samples were retrieved from Northern Irish wild fallow deer abomasa. Individual specimens were subject to a species-specific PCR analysis for common sheep and cattle GIN species with ITS-2 sequence analysis to validate species identities. In addition, the beta-tubulin gene was subject to sequencing to identify benzimidazole (BZ) resistance markers.

**Results:**

ITS-2 sequencing revealed *O. leptospicularis* and *S. asymmetrica*, but species-specific PCR yielded false-positive hits for *H. contortus*, *Teladorsagia circimcincta, Trichostrongylus axei, T. colubriformis, T. vitrinus* and *Ostertagia ostertagi*. For beta-tubulin, *O. leptospicularis* and *S. asymmetrica* yielded species-specific sequences at the E198 codon, but no resistance markers were identified in either species at positions 167, 198 or 200 of the coding region.

**Discussion:**

From this report, no GIN species of significance in livestock were identified among Northern Ireland fallow deer. However, false-positive PCR hits for sheep and cattle-associated GINs is concerning as the presence of deer species in livestock areas could impact both deer and livestock diagnostics and lead to overestimation of both GIN burden in deer and the role as of deer as drivers of these pathogens. ITS-2 sequences from both *O. leptospicularis* and *S. asymmetrica* show minor sequence variations to geographically distinct isolates. AR has been noted among GINs of deer but molecular analyses are lacking for GINs of wildlife. In producing the first beta-tubulin sequences for both *O. leptospicularis* and *S. asymmetrica*, we report no BZ resistance in this cohort.

**Conclusions:**

This work contributes to genetic resources for wildlife species and considers the implications of such species when performing livestock GIN diagnostics.

**Graphical Abstract:**

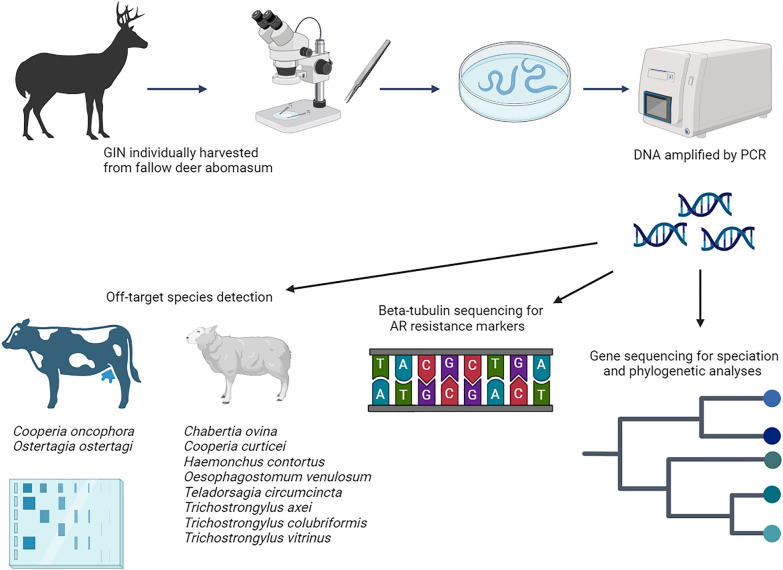

**Supplementary Information:**

The online version contains supplementary material available at 10.1186/s13071-024-06147-2.

## Background

Across the northern hemisphere, including Great Britain and Northern Ireland (NI), wild deer populations are increasing [1–3], resulting in deer encroachment onto livestock pastures and increased contact with livestock [4–6]. This raises questions about the impact of deer on livestock farming, especially given that deer species are susceptible to a number of economically significant livestock parasites, principally gastrointestinal nematodes (GINs) [5–11].

GIN infections of ruminants are complex, with many species capable of infecting a range of hosts across livestock and wildlife barriers [5, 7, 11]. For instance, pathogenic species of livestock such as *Teladorsagia circimcincta* [11] and *Haemonchus contortus* have been reported in natural infections of wild cervid species [8, 12–14]. Conversely, wildlife GIN species can cause infections in livestock such as *Ostertagia leptospicularis* in sheep [15–19] and cattle [17].

In the UK and Republic of Ireland alone, GINs cause an estimated €280 million in production losses annually, with a further €42 million spent on anthelmintic control measures [20]. Use of anthelmintics is by far the most common GIN control measure utilised in commercial livestock settings, but long-term reliance on a few classes of compounds has led to the emergence and spread of anthelmintic resistance among livestock GIN populations [21–29]. As such, AR is a major problem within the livestock industry and threatens closure of farms in areas where no anthelmintic control approaches are effective [30–32]. However, there are limited analyses of AR traits among GIN species of wild deer.

AR GIN populations are known to exist amongst farmed deer, for instance benzimidazole-resistant *H. contortus* have been reported [14]. Reduced anthelmintic efficacy has also been demonstrated in more cervid-restricted species, including in *O. leptospicularis, Spiculopteragia spiculoptera* and *S. asymmetrica*, for which no molecular markers for resistance are known [33–38]. Unlike farmed deer, wild cervids are not routinely treated with anthelmintics; therefore, carriage of resistant genotypes may be seen as indicative of interspecies contact with livestock or farmed deer [5, 7, 38–40]. However, AR traits are also known to occur naturally in free living nematodes such as *Caenorhabditis elegans* [41]. Additionally, wildlife may be exposed to low levels of anthelmintics in the environment such as in wastewater or on pasture [42, 43]. Therefore, GINs of cervids may also have developed resistance traits and warrants investigation.

In the face of widespread AR-GINs in livestock farming systems, approaches to improve diagnostics have been made partly to enable sustainable use of anthelmintics and to better our knowledge of GIN infections spatiotemporally [44, 45]. It is also critical to differentiate GIN species to provide an appropriate anthelmintic treatment [46]. However, morphological diagnostic approaches require expert knowledge and time-consuming methods to accurately diagnose specific species [47]. Currently, a number of DNA-based diagnostic approaches are in use or under development to detect and enumerate the presence of different GIN species based on species-specific markers that can detect individual GIN parasites by PCR [48–55], loop-mediated isothermal amplification (LAMP) [52, 56] or ITS-2 meta-barcoding [57, 58]. Methods have also been developed for the detection of deer and other wildlife GINs in both wildlife and livestock contexts [39, 46, 59, 60]. However, as these emerging technologies evolve, DNA databases need to keep pace with accurate and comprehensive data to ensure clarity of speciation [46]. A lack of knowledge remains pertaining to the accuracy and utility of DNA diagnostic tools in the presence of wildlife GIN species.

In NI, deer are the most prominent wild ruminants with fallow (*Dama dama*), red (*Cervus elaphus*) and Japanese sika (*Cervus nippon*) present, all of which are susceptible to GINs of livestock [61, 62]. However, GIN infections and AR in wild deer have gone largely under-investigated at both local and international scales. In this study, abomasa samples were obtained from three populations of Northern Ireland’s most abundant deer species, fallow deer, for nematode speciation and subsequent genetic analysis for evidence of resistance to widely used benzimidazole (BZ) anthelmintics. In addition, we cross-validate known PCR diagnostic primer sets against deer-specific species identified in this study to determine the potentiality of wildlife species yielding false-positive PCR results for livestock diagnostics.

## Methods

### Wild fallow deer specimen retrieval and DNA extraction

Seven fallow deer (*Dama dama*) were culled by trained stalkers, in adherence with Northern Ireland legislation, across three locations, Co. Antrim (Randalstown Forest), Co. Down (Tollymore Forest) and Co. Londonderry (Ardkill), during the legal open season (November 2019). Randalstown Forest is a 172 ha mixed conifer and broadleaf woodland situated at the northern edge of Lough Neagh. Tollymore Forest is a 630 ha mixed conifer and broadleaf woodland at the foot of the Mourne Mountains in the southeast of the country and Ardkill has several small forested areas situated in the north west of the country. Each of these woodland areas is surrounded by cattle and sheep pasture.

Adult nematodes were washed from abomasum tissue, cleaned in ddH_2_O and then stored in 70% EtOH at − 20 °C. Individual worms were washed thrice in 0.5 ml lysis buffer [100 mM KCl, 20 mM Tris pH 8, 2.5 mM MgCl_2_, 0.9% IGEPAL^®^ CA-630 (Sigma-Aldrich, St. Louis, MO, USA), 0.9% Tween-20 (Sigma-Aldrich) and 0.02% gelatin, see nemabiome.ca website for details] and then transferred by pipette to an ethanol-cleaned microscope slide under a stereomicroscope and bisected in individual droplets of lysis buffer by bleach cleaned scalpel to break the cuticle. Worm pieces were transferred to individual tubes by bleach-cleaned no. 5 forceps with one sample taken per worm. DNA lysis was adapted from the Nemabiome protocol [57] with each sample digested in 100 µl lysis buffer and 4 µl proteinase K (0.8 mg/ml final concentration, NEB, Ipswich, MA, USA), which was vortexed, centrifuged and then digested overnight or at 56 °C in a water bath. Proteinase K was inactivated at 95 °C for 20 min and samples were stored at − 20 °C (20 mg/ml solution).

### Species-specific PCR screening

PCR reactions were performed with GoTaq^®^ G2 Hot Start Taq Polymerase (Promega, Madison, WI, USA) at recommended concentrations (2.5 mM MgCl2, 0.2 mM dNTPs (PCRbio, London, UK) and 0.4 µM of each primer). Previously published primers and the recommended annealing temperatures used are listed in Additional file 2: Table S1 [49, 63–70]. For all reactions, 25 μl volumes were used with 1 μl of DNA lysate per reaction or nuclease-free water for no template controls. For all PCR reactions, a 95 °C/2 min initial denaturation step and a final extension at 72 °C for 5 min was performed. For Bisset et al. primers [49], touchdown PCR conditions were used including: 12 cycles of 95 °C denaturation for 15 s, 60 °C annealing (with 0.5 °C decline per cycle), 72 °C extension for 30 s, followed by 25 cycles of 95 °C for 15 s, 54 °C for 15 s and 72 °C for 30 s as previously described. For other primers, 35 cycles of 95 °C denaturation for 15 s, 52–58 °C annealing for 15 s and 72 °C extension for 30 s were used, with as previously described. See Additional file 2: Table S1 for specific details. PCR products were visualized by precast 1 × concentration SYBRSafe (Thermo Fisher, Waltham, MA, USA) on 1.3% agarose gel electrophoresis in TAE buffer.

### Sanger sequencing

PCR was performed with Q5^®^ High-Fidelity DNA Polymerase (New England Biolabs) per manufacturer’s instructions. For speciation, ITS-2 NC1-forward (5′-ATTGCGCCATCGGGTTCATTCC-3′) and NC2-reverse (5′-TTAGTTTCTTTTCCTCCGCT-3′) primers were used [67]; 1 μl of DNA lysate was used per 25 μl reaction. Cycling conditions included an initial denaturation (98 °C/30 s), 35 cycles (98 °C/15 s, 52 °C/30 s, 72 °C/30 s) and final extension (72 °C/2 min). For beta-tubulin sequences, previously designed forward (5′-NNNACGCACTCTTTGGGAGGAGG-3′) and reverse (5′-NNNTGTGAGTTTTAGTGTGCGGAAG-3′) primers were used that span exons 4 and 5 and intron 4 of the beta-tubulin isotype 1 gene [71]; 1 μl of DNA lysate was used per 25 μl reaction. Cycling conditions included an initial denaturation (98 °C/30 s), 35 cycles (98 °C/15 s, 55 °C/30 s, 72 °C/35 s) and final extension (72 °C/2 min). PCR products were assessed by 1.3% agarose gel electrophoresis with single bands purified by Wizard^®^ SV Gel and PCR Clean-Up System (Promega) and assessed by NanoDrop^®^ ND-1000 (Thermo Fisher). Purified PCR products were sequenced by Eurofins TubeSeq service and assessed for quality and base-calling using Bioedit 7.2.5 software (University of North Texas).

### Phylogenetic analyses of ITS-2 sequences

Individual ITS-2 sequences were speciated in Geneious Prime^®^ (2023.1.1 Build 2023-04-03) using the ‘identify organism’ tool to BLAST against the nemabiome ITS-2 database [72]. Sequences and hits with the highest overall sequence query coverage and identity are shown in Additional file 3: Table S2. A single consensus sequence was produced from MUSCLE (PPP) aligned sequences and submitted GenBank for *O. leptospicularis* (Genbank accession no. OR284984) and *S. asymmetrica* (Genbank accession no. OR284985).

Phylogenetic trees were produced using previously documented protocols for GIN ITS-2 phylogenetics [71] using Geneious Prime^®^. Briefly, ingroup and outgroup taxa were selected and aligned by MUSCLE (PPP) with trees produced using Jukes-Cantor model computed with 10,000 bootstrap replicates and rooted on *T. axei*. Comparison species were selected from known species in wild ruminants [11] alongside other representative Haemonchidae with random representative sequences from the nemabiome ITS-2 database [72]. To compare individual samples, the same method was used except consensus sequences from ingroup taxa were selected to minimize tree size while still comparing sample variance.

### Analysis of beta-tubulin sequences

Sanger sequencing was performed as described above for a sub-set of individual specimens. Individual sequences were assessed for the presence of four known markers of BZ resistance, F167Y (TTC/TAC), E198A (GAR/GCA) or E198L (GAR/TTA) and F200Y (TTC/TAC) [58, 71, 73, 74]. Aligned and consensus sequences are shown in Additional file 4: File S1. Consensus sequences were submitted to GenBank for *O. leptopiscularis* (PP077401) and *S. asymmetrica* (PP077402).

### Data presentation

Unless otherwise stated, original data were tabulated in Microsoft Excel with figure layouts generated in Adobe^®^ Illustrator 2023 (Adobe Inc).

## Results

### Assessment of gastrointestinal nematodes from fallow deer necropsies

To determine whether deer in Northern Ireland harbour sheep- or cattle-associated GINs, adult worms were collected from abomasa and subjected to Sanger sequencing molecular speciation using NC1/NC2 pan-nematode primers (Additional file 2: Table S1). In abomasa *O. leptospicularis* and *S. asymmetrica* coinfections were identified in 4/7 of individuals, with more *S. asymmetrica* identified overall (Table 1). No other GIN species were identified.

**Table 1:** Tally of gastrointestinal nematodes identified from deer necropsies

Deer ID	Location	*Ostertagia leptospicularis*	*Spiculopteragia asymmetrica*	Total
A.142301	Ardkill	4	5	9
B.140048	Tollymore	0	14	14
C.142323	Randalstown	11	4	15
D.140049	Tollymore	0	14	14
E.142322	Randalstown	0	2	2
F.140032	Randalstown	3	3	6
G.14234	Randalstown	4	11	15
Total		22	53	75
Proportion of infected deer		4/7	7/7	

**Fig. 1:**
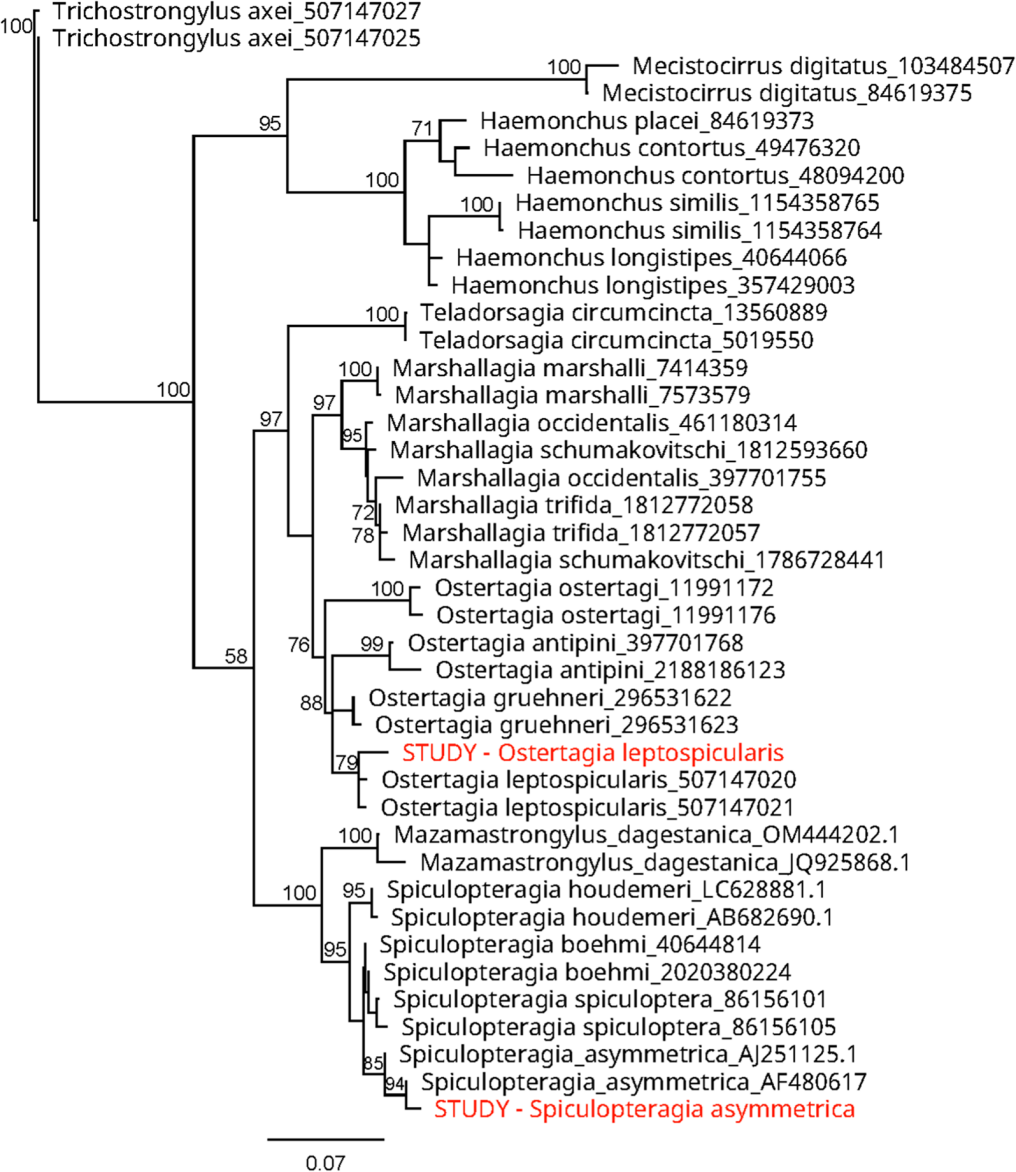
ITS-2 phylogenetic neighbour-joining tree from isolated samples compared to parasites of wild ruminants and similar Haemonchidae species. Random representative sequences selected from the Nemabiome ITS-2 database for reference species. Sequences aligned by MUSCLE (PPP) and organised by the Jukes-Cantor distance model (10,000 bootstrap replicates) with *Trichostrongylus axei* used as an outgroup. Tree displays species and accession number used with consensus sequences from in-study specimens colour coded. Bootstrap values > 70% shown

Inter-sample sequence variation was minimal for *O. leptospicularis* (96.43–100% identity, median = 100%) and *S. asymmetrica* (94.62–100% identity, median = 100%) and all individual samples clustered when compared to other Haemonchidae (Additional file 1: Fig. S1). Consensus sequences for each species yielded mildly distinct variations from known isolates (Fig. 1) and may represent Ireland-specific variants for both *O. leptospicularis* and *S. asymmetrica*.

### Cross-validation of sheep and cattle GIN species-specific PCR primers against deer parasites

**Fig. 2:**
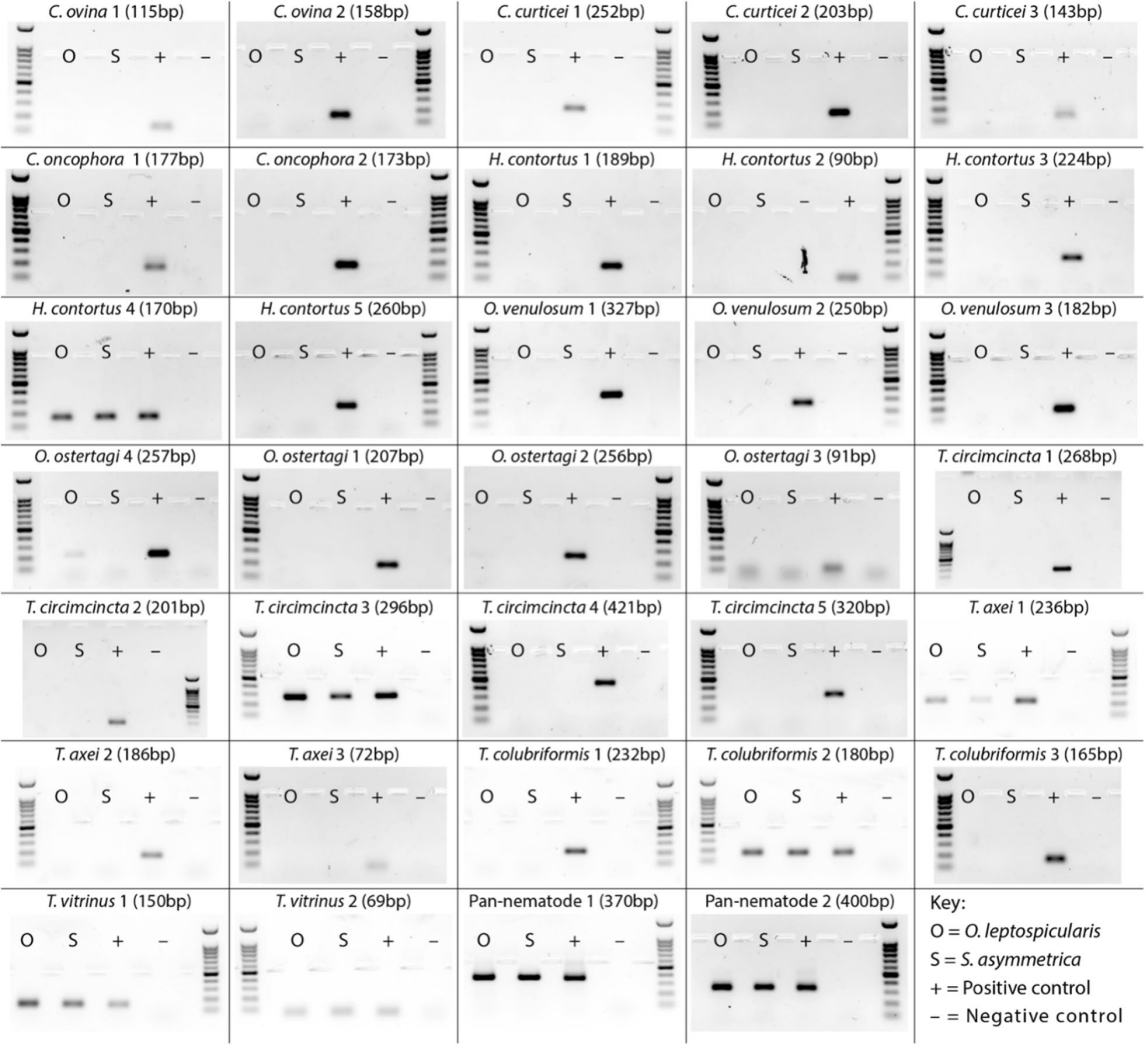
Species-specific PCR screen with false-positive hits against *Ostertagia leptospicularis* and *Spiculopteragia asymmetrica* specimen pools. Primer sets shown as outlined in Additional file 2: Table S1 and results summarised in Table 2, with target species and expected PCR product sizes (in brackets) tested against O = *O. leptospicularis*, S = *S. asymmetrica*, (+) = positive control DNA, and (−) = negative no template control. Ladder = 100 bp with double-intensity 500-bp band

An array of species-specific primers have previously been designed to detect sheep and cattle GIN species. To determine whether *O. leptospicularis* or *S. asymmetrica* yield cross-contamination of sheep and cattle GINs, we performed a PCR screen from published primers [49, 66]. We found that primers for *H. contortus, T. circimcincta, T. axei, T. colubriformis* and *T. vitrinus* cross-reacted with both *O. leptospicularis* and *S. asymmetrica*, while a primer set for *O. ostertagi* cross reacted with *O. leptospicularis* (Table 2, Additional file 2: Table S1, Fig. 2). Overall, the majority of primer sets worked as expected against these wildlife species (Table 2), but false-positive reactions produced single PCR product bands that were indistinguishable in size from positive controls (Fig. 2). One of the primer sets tested for *T. circimcincta* (‘TeciFd3’) has previously documented cross-reactivity to *O. leptospicularis* [49].

**Table 2:** False-positive PCR hits from species-specific primer sets

Host	Target species	Primers tested*	*Ostertagia leptospicularis* hits?**	*Spiculopteragia asymmetrica* hits?**
Cattle	*Cooperia onchophora*	2		
Cattle	*Ostertagia ostertagi*	4	1	
Sheep	*Chabertia ovina*	2		
Sheep	*Cooperia curticei*	3		
Sheep	*Haemonchus contortus*	5	1	1
Sheep	*Oesophagostomum venulosum*	3		
Sheep	*Teladorsagia circumcincta*	5	1	1
Sheep	*Trichostrongylus axei*	3	1	1
Sheep	*Trichostrongylus colubriformis*	3	1	1
Sheep	*Trichostrongylus vitrinus*	2	2	2
*na*	Pan-nematode (+ control)	2	2	2

*Primers shown in Additional file 2: Table S1 and representative result gel images shown in Fig. 2. **Single PCR products indistinguishable in size compared to positive controls

### Beta-tubulin sequences from *O. leptospicularis* and *S. asymmetrica*

A total of 30 sequences from *O. leptospicularis* (*n* = 15) and *S. asymmetrica* (*n* = 15) were performed using previously published primers [75]. All samples tested yielded susceptible sequences at positions 167 (TTC), 198 (GAG/GAA) and 200 (TTC) (Additional file 4: File S1). For all *O. leptospicularis* GAG sequences were found at position 198, while for all *S. asymmetrica* GAA was found at position 198. Sequences had high identities across individuals with 203/226 matching bases for *O. leptospicularis* (91.11–100% pairwise identity). For *S. asymmetrica* 219/230 bases matched (96.52–100% range in pairwise identity) (Additional file 1: File S1).

## Discussion

Little is known relating to populations of GINs among Northern Irish fallow deer and whether these GINs harbour AR traits. To elucidate the status of GIN presence among wild fallow deer in Northern Ireland, we investigated deer carcasses from individuals covering three counties inhabited by over two-thirds of the total NI sheep population (67%) and over half the cattle population (53%) [76]. In this study we identified two species commonly associated with cervids, *O. leptospicularis* and *S. asymmetrica* among abomasa of seven deer. Both of these species are frequently reported in cervids and occasionally in sheep and cattle, but molecular resources for these species are lacking. To add to available resources, we provide consensus ITS-2 Sanger sequences from a total of 22 *O. leptospicularis* (OR284984) and 53 *S. asymmetrica* (OR284985), with individual sequences presented in Additional file 3: Table S2. In addition, we provide the first reported beta-tubulin sequences for both species (PP077401-PP077402) to our knowledge.

In improving genetic resources for these species, we note the importance of including wildlife and less common species in development of molecular diagnostic assays, such as for monitoring the presence of livestock GINs. Interest in molecular diagnostics (particularly PCR based diagnostics) of GINs and other helminths is broad and growing. Methods for detection vary and rely on standardization with available parasite materials. In principle, PCR diagnostics utilise conserved regions of parasite genomes, such as the ITS-2 region of rDNA with species- or genus-specific primers or probes designed at variable sites [49–55]. PCR-based amplification yields fluorescence from specific probes in loop-mediated isothermal amplification (LAMP) [52, 56], Q-PCR [54, 68, 77] and ddPCR [78], size-specific products in endpoint PCR and semi-quantitative PCR [48, 49] or products with specific melt curves in high-resolution melt curve analyses [48, 55]. Molecular monitoring of GINs can utilize a number of genetic sources such as: (1) adult worms from necropsies [55, 79], (2) faecal DNA [48, 79], (3) isolated eggs from faeces [77], (4) cultured larvae [53, 57], (5) pasture larvae [80] and (6) environmental DNA [81]. However, validation of probe specificity has typically not taken wildlife species into consideration, although multiple studies have been mindful to detect wildlife species [39, 46, 59, 60]. In this study, we test *O. leptospicularis* and *S. asymmetrica* DNA from fallow deer in NI against a number of livestock GIN specific primers using endpoint PCR with size specific primers.

While the majority of primers tested produced no cross-reactivity against either species, we generated false-positive hits at expected product sizes for *H. contortus* (1/5 primers), *O. ostertagi* (1/4 primers), *T. circimcincta* (1/5 primers), *T. axei* (1/3 primers), *T. colubriformis* (1/3 primers) and *T. vitrinus* (2/2 primers). We should note that the *T. circimcincta* primer set (TeciFd3) is known to cross-react to *O. leptospicularis* [49] and so this finding is not surprising. However, cross-reactivity with both *O. leptospicularis* and *S. asymmetrica* is concerning as it raises questions about potential other wildlife species that could contaminate molecular diagnostic approaches. While closely related species such as *O. ostertagi* might be expected to yield false-positive hits, the variety of species detected is concerning since there is potential for *O. leptospicularis* and *S. asymmetrica* DNA to be present in any number of sources.

With deer co-grazing on pasture becoming increasingly prominent, deer GINs may be increasingly deposited on pastures where the eggs or larvae can contaminate faeces collected from grass for faecal egg counts and molecular diagnostics, contaminate pasture larvae counts or contaminate environmental DNA collections. In addition, as *O. leptospicularis* can cause infections in both cattle and sheep, molecular misidentifications can also be made from worms collected at necropsy. PCR diagnostics have previously been performed on adult worms collected from roe deer, but such studies are robust and have included additional checks such as ITS-2 sequencing to confirm species identity [13]. However, whether PCR alone is sufficient to identify livestock parasites remains to be determined.

Cervid species such as *O. leptospicularis* may also pose a threat to the performance of cattle [17] and sheep [15, 19] and are capable of altering intestinal pH [16]. Interestingly, distinct *O. leptospicularis* populations in cattle have been found and possibly adapt to local hosts [10]. *Spiculopteragia asymmetrica* is also identified in some farmed ruminants such as mouflons [59] but may have capacity to overspill into more livestock species in areas where wildlife are declining or where shared grazing is increasing [5, 82]. Infections of *O. leptospicularis* in livestock may also produce sterile hybrid offspring with *O. ostertagi* [18]. This in turn can result in false-positive detection of sheep or cattle parasites by PCR from pasture larvae or pasture collected faecal samples since deer faeces will contaminate the pasture. Furthermore, sterile hybrid infections will also go undetected in faecal egg counts as these have reduced or ablated egg production [18]. However, both *O. leptospicularis* and *S. asymmetrica* cross-reacted with the same off-target primer sets, with the exception of *O. ostertagi* which did not yield a false-positive hit for *S. asymmetrica.* As such, it is probable that primers can be effectively designed even in the presence of off-target species from wildlife contamination.

Beyond ITS-2 markers we also provide the first beta-tubulin sequences for both *O. leptospicularis* and *S. asymmetrica*. Monitoring resistance markers in wildlife is crucial to determine the extent of overlap between anthropogenic and wild habitats and has been applied for species with wild and farmed populations such as bison [75]. A number of single-nucleotide polymorphism (SNP) sites that confer resistance to BZ are known for other GIN species [83], but have yet to be identified in wildlife restricted species. Here we find that beta-tubulin sequences vary in intronic areas but predicted protein coding regions match with predicted and known other nematode protein sequences. No AR SNPs were detected for any samples indicating that despite the small sample size collected, there is no evidence for AR among these species in wild cervids in Northern Ireland currently. While we did not find any evidence of BZ resistance in our sample cohort, we demonstrate that effective monitoring of BZ resistance sites by sequencing is possible as has been reported for livestock species [71]. These sequences also provide a means to develop PCR monitoring for BZ SNPs as previously developed for livestock species [31].

Despite the lack of resistance markers present in the current study, monitoring for AR among wildlife is critical to determine potential toxicological impacts of livestock–wildlife interfaces. For instance, widespread use of anthelmintics has led to ecological contamination in a wide variety of substrates including water [84, 85], soil [42, 86] and plants [42, 86]. However, most common anthelmintics such as BZ, macrocyclic lactones and levamisole are photolabile and will degrade in sunshine, and they may not pose a significant direct effect in sunny summer conditions [87]. Nevertheless, build-up of anthelmintic residues on pasture can lead to auto-dosing of untreated animals [42]. In this case, sheep dosed with albendazole were allowed to excrete on fodder plants. Later feeding of fodder plants from contaminated pasture led to albendazole detected in untreated sheep [42]. As such, it is entirely possible for wildlife species to be similarly impacted if co-grazing in dirty pasture.

Overall, this dataset provides a baseline to monitor BZ resistance markers in *O. leptospicularis* and *S. asymmetrica* as well as details of ITS-2 sequences for improved species-specific molecular diagnostics of livestock GINs.

## Conclusions

We provide a case report of abomasal worms from seven fallow deer culled in regions with relatively high sheep ownership. Individual adult worms were isolated and speciated, revealing *O. leptospicularis* and *S. asymmetrica*. To our surprise, both species yielded cross-reactivity to a number of livestock GIN species using previously published species-specific PCR primers, a result that highlights the need to consider wildlife species when designing molecular diagnostics. Since coinfections of livestock and wildlife are diverse and often overlap, it is critical to build upon molecular resources for wildlife species which are a neglected part of the ecology of parasite dispersal in shared environments.

### Supplementary Information

**Additional file 1. File S1** -  Multiple sequence alignments and identity matrices for Beta tubulin isoform 1 sequences.

**Additional file 2. Table S1** - Primers and cycling conditions used for PCR.

**Additional file 3. Table S2** - ITS-2 Sanger sequences for each individual analysed.

**Additional file 4. Supplementary Materials** - Supplementary Figures and Table headings.

## Data Availability

Baseline data from nematode species counts, PCR results and DNA sequences are presented in the manuscript and supplemental materials.

## Abbreviations

AR: Anthelmintic resistance
GIN: Gastrointestinal nematode parasite
ITS-2: Ribosomal internal transcribed spacer 2 region
NI: Northern Ireland
PCR: Polymerase chain reaction
