# Supplementary material for: A molecular assessment of Ostertagia leptospicularis and Spiculopteragia asymmetrica among wild fallow deer in Northern Ireland and implications for false detection of livestock-associated species
Source: Parasit Vectors. 2024 Mar 18;17:141. doi: 10.1186/s13071-024-06147-2 (PMC10949651; doi:10.1186/s13071-024-06147-2)
Supplement: Supplementary file 1 — Additional file 1. File S1 - Multiple sequence alignments and identity matrices for Beta tubulin isoform 1 sequences. [file 13071_2024_6147_MOESM1_ESM.docx]

# Supplementary Materials for

# “A molecular assessment of *Ostertagia leptospicularis* and *Spiculopteragia asymmetrica* infections of Northern Ireland Fallow deer with a note on false positive PCR diagnostics”

## Authors:

Maggie Lyons^ab†^, Paul M. Airs^a†^, Tony Brown^a^, Angela Lahuerta-Marin^b^, Eric. R. Morgan^a^*

**Affiliation:** ^a^School of Biological Sciences, Queen’s University Belfast, 19 Chlorine Gardens, Belfast, BT9 5DL, UK; ^b^Agri-food & Biosciences Institute Northern Ireland, 12 Stoney Road, Belfast, Co Antrim, BT4 3SD, UK.

* Corresponding author: [eric.morgan@qub.ac.uk](mailto:eric.morgan@qub.ac.uk)

# Supplementary Figures


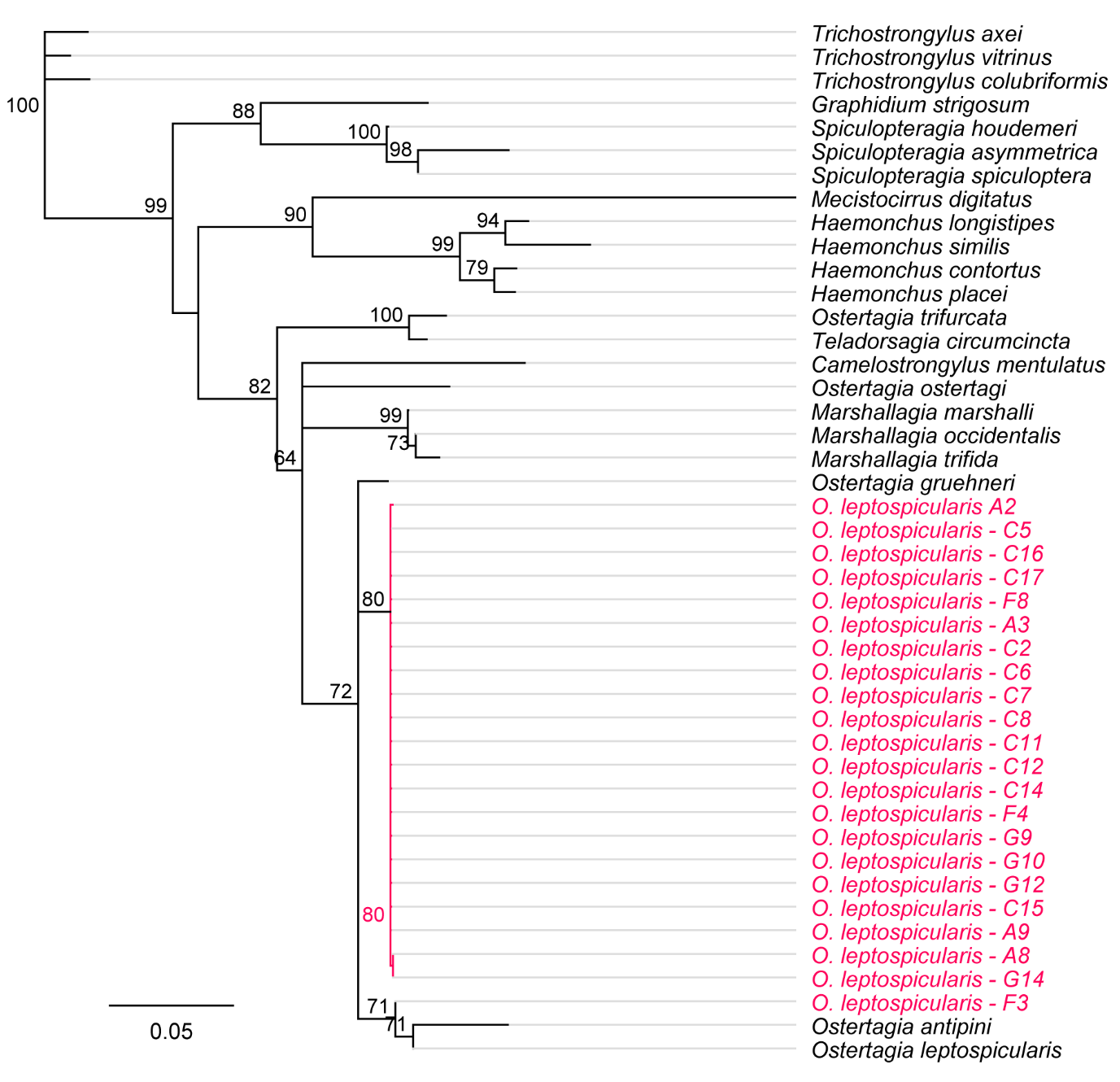


**Fig. S1: ITS-2 phylogenetic neighbour-joining tree from individual *O. leptospicularis* samples compared to parasites of wild ruminants and similar Haemonchidae species.** Consensus sequences from selected species generated from the Nemabiome ITS-2 database. Sequences aligned by MUSCLE (PPP) and organised by the Jukes-Cantor distance model (10,000 bootstrap replicates) with *T. axei* used as an outgroup. Tree displays species and accession number used with consensus sequences from in-study specimens colour coded. Bootstrap values >70% shown.


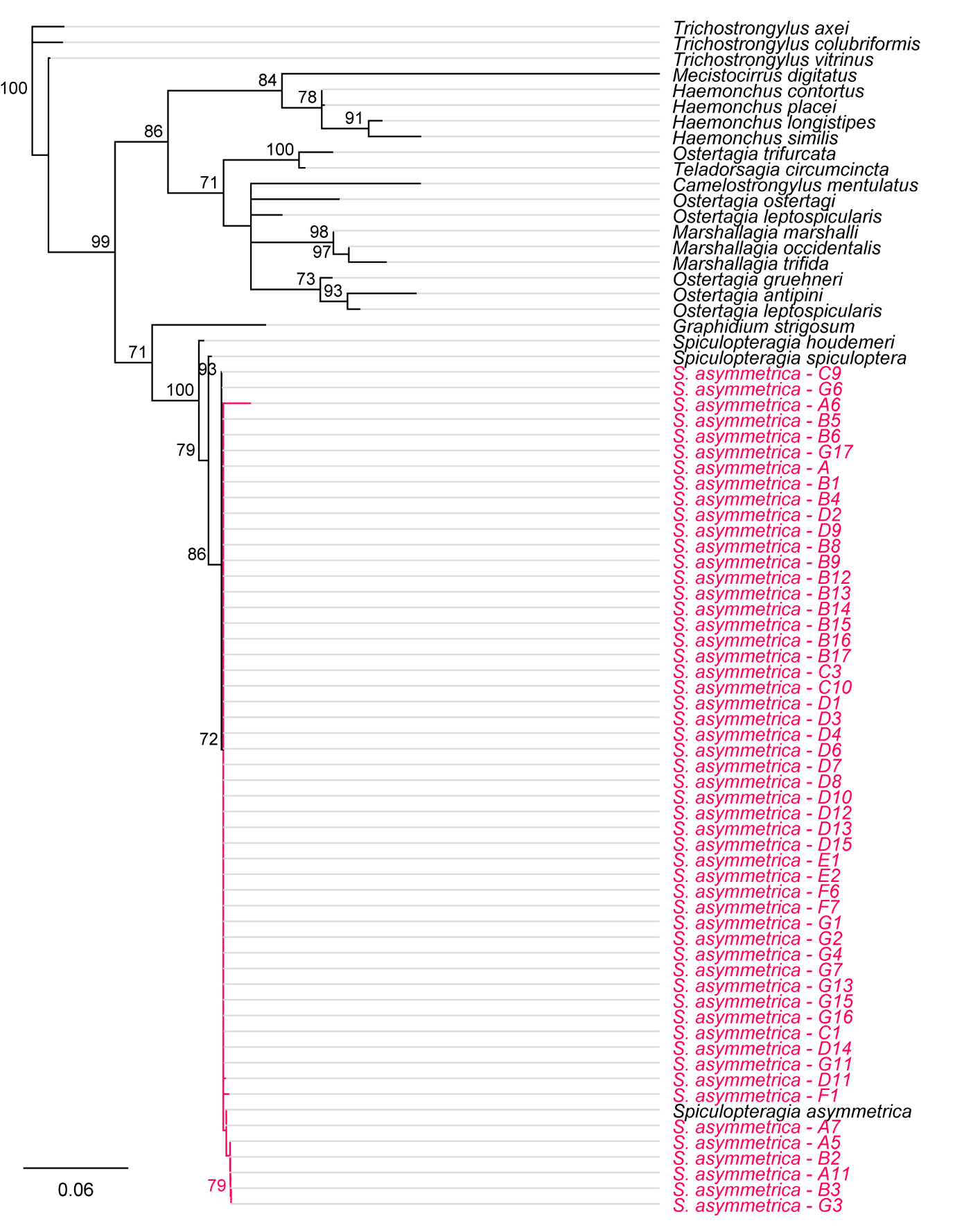


**Fig. S2: ITS-2 phylogenetic neighbour-joining tree from individual *S. asymmetrica* samples compared to parasites of wild ruminants and similar Haemonchidae species.** Consensus sequences from selected species generated from the Nemabiome ITS-2 database. Sequences aligned by MUSCLE (PPP) and organised by the Jukes-Cantor distance model (10,000 bootstrap replicates) with *T. axei* used as an outgroup. Tree displays species and accession number used with consensus sequences from in-study specimens colour coded. Bootstrap values >70% shown.

# Supplementary Tables

[file uploaded separately]

## Table S1: Primers and cycling conditions used for PCR.

† Touchdown PCR conditions denote a starting annealing temperature of 60^o^C with a delta of -0.5^o^C for 12 cycles, reaching 54^o^C, before 25 more cycles at 54^o^C. * Hits defined as single PCR products indistinguishable in size compared to positive controls.

Bold primers are pan-nematode used for speciation. Representative gel electrophoresis images shown in Fig. 2.

[file uploaded separately]

## Table S2: ITS-2 Sanger sequences for each individual analysed.

## Sequences from individual adult worms taken from deer listed in Table 1 with species typing from BLAST analysis (see materials and methods).

[file uploaded separately]

**File S1: Beta-tubulin Sanger sequences for each individual analysed.**

## Sequences from individual adult worms taken from deer listed in Table 1 with species typing from BLAST analysis (see materials and methods).
