# Supplementary material for: A molecular assessment of Ostertagia leptospicularis and Spiculopteragia asymmetrica among wild fallow deer in Northern Ireland and implications for false detection of livestock-associated species
Source: Parasit Vectors. 2024 Mar 18;17:141. doi: 10.1186/s13071-024-06147-2 (PMC10949651; doi:10.1186/s13071-024-06147-2)
Supplement: Supplementary file 4 — Additional file 4. Supplementary Materials - Supplementary Figures and Table headings. [file 13071_2024_6147_MOESM4_ESM.docx]

**File S1 - Multiple sequence alignments and identity matrices for *Beta tubulin isoform 1* sequences**

Sequences shown below with known positions with resistant mutations to benzimidazoles including P167 (**TTC**), P198 (**GAG**/**GAA**), and P200 (**TTC**) shown in bold underline font. Consensus shows protein coding and intronic regions. Exons highlighted.

***Ostertagia leptospicularis Beta-tubulin isoform 1* consensus sequence**

**TTC**TCAGTTGTTCCTTCGCCTAAGGTAATATACCCCAACAGTCGCCTCATTTTGAGAGTTTTATGTACAGACTCTCGGGATGCTGCTCATTATCTCAACTTCGGTACTTAACGCTAACAATTAAATATGCGATGGAATTTTGCTTATAGGTCTCCGATACCGTTGTGGAGCCCTACAACGCCACTCTTTCTGTCCACCAGTTGGTAGAAAATACTGAT**GAG**ACG**TTC**

***Ostertagia leptospicularis* Percent Identity Matrix - created by Clustal2.1**

Os_lep_F3 100.00 91.56 91.56 91.56 91.56 91.11 91.15 91.59 91.59 91.59 91.15 91.59 91.59 92.04 92.00

Os_lep_A2 91.56 100.00 100.00 100.00 100.00 99.56 98.23 98.67 98.67 98.67 98.23 98.67 99.12 99.12 99.56

Os_lep_C5 91.56 100.00 100.00 100.00 100.00 99.56 98.23 98.67 98.67 98.67 98.23 98.67 99.12 99.12 99.56

Os_lep_F4 91.56 100.00 100.00 100.00 100.00 99.56 98.23 98.67 98.67 98.67 98.23 98.67 99.12 99.12 99.56

Os_lep_G14 91.56 100.00 100.00 100.00 100.00 99.56 98.23 98.67 98.67 98.67 98.23 98.67 99.12 99.12 99.56

Os_lep_C16 91.11 99.56 99.56 99.56 99.56 100.00 98.67 99.12 99.12 99.12 98.23 98.67 98.67 98.67 99.11

Os_lep_C7 91.15 98.23 98.23 98.23 98.23 98.67 100.00 99.56 99.56 99.56 98.68 99.12 98.68 99.12 99.12

Os_lep_C2 91.59 98.67 98.67 98.67 98.67 99.12 99.56 100.00 100.00 100.00 99.12 99.56 99.12 99.56 99.56

Os_lep_C17 91.59 98.67 98.67 98.67 98.67 99.12 99.56 100.00 100.00 100.00 99.12 99.56 99.12 99.56 99.56

Os_lep_G9 91.59 98.67 98.67 98.67 98.67 99.12 99.56 100.00 100.00 100.00 99.12 99.56 99.12 99.56 99.56

Os_lep_A8 91.15 98.23 98.23 98.23 98.23 98.23 98.68 99.12 99.12 99.12 100.00 99.56 98.68 99.12 99.12

Os_lep_A9 91.59 98.67 98.67 98.67 98.67 98.67 99.12 99.56 99.56 99.56 99.56 100.00 99.12 99.56 99.56

Os_lep_C8 91.59 99.12 99.12 99.12 99.12 98.67 98.68 99.12 99.12 99.12 98.68 99.12 100.00 99.56 100.00

Os_lep_C11 92.04 99.12 99.12 99.12 99.12 98.67 99.12 99.56 99.56 99.56 99.12 99.56 99.56 100.00 100.00

Os_lep_F8 92.00 99.56 99.56 99.56 99.56 99.11 99.12 99.56 99.56 99.56 99.12 99.56 100.00 100.00 100.00

***Ostertagia leptospicularis* CLUSTAL O(1.2.4) multiple sequence alignments**

Os_lep_F3 **TTC**TCAGTTGTTCCTTCGCCTAAGGTAATATACCCCTACAGTTGCCTCATTTTGAGACTT 60

Os_lep_A2 **TTC**TCAGTTGTTCCTTCGCCTAAGGTAATATACC-CCAAAGTCGCCTCATTTTGAGAGTT 59

Os_lep_C5 **TTC**TCAGTTGTTCCTTCGCCTAAGGTAATATACC-CCAAAGTCGCCTCATTTTGAGAGTT 59

Os_lep_F4 **TTC**TCAGTTGTTCCTTCGCCTAAGGTAATATACC-CCAAAGTCGCCTCATTTTGAGAGTT 59

Os_lep_G14 **TTC**TCAGTTGTTCCTTCGCCTAAGGTAATATACC-CCAAAGTCGCCTCATTTTGAGAGTT 59

Os_lep_C16 **TTC**TCAGTTGTTCCTTCGCCTAAGGTAATATACC-CCAAAGTCGCCTCATTTTGAGAGTT 59

Os_lep_C7 **TTC**TCAGTTGTTCCTTCGCCTAAGGTAATATACCCCAACAGTCGCCTCATTTTGAGAGTT 60

Os_lep_C2 **TTC**TCAGTTGTTCCTTCGCCTAAGGTAATATACCCCAACAGTCGCCTCATTTTGAGAGTT 60

Os_lep_C17 **TTC**TCAGTTGTTCCTTCGCCTAAGGTAATATACCCCAACAGTCGCCTCATTTTGAGAGTT 60

Os_lep_G9 **TTC**TCAGTTGTTCCTTCGCCTAAGGTAATATACCCCAACAGTCGCCTCATTTTGAGAGTT 60

Os_lep_A8 **TTC**TCAGTTGTTCCTTCGCCTAAGGTAATATACCCCAACAGTCGCCTCATTTTGAGAGTT 60

Os_lep_A9 **TTC**TCAGTTGTTCCTTCGCCTAAGGTAATATACCCCAACAGTCGCCTCATTTTGAGAGTT 60

Os_lep_C8 **TTC**TCAGTTGTTCCTTCGCCTAAGGTAATATACCCCAANAGTCGCCTCATTTTGAGAGTT 60

Os_lep_C11 **TTC**TCAGTTGTTCCTTCGCCTAAGGTAATATACCCCAACAGTCGCCTCATTTTGAGAGTT 60

Os_lep_F8 **TTC**TCAGTTGTTCCTTCGCCTAAGGTAATATACCCCAA-AGTCGCCTCATTTTGAGAGTT 59

********************************** * * *** ************** **

Os_lep_F3 G-WAGTACAGGTTTTCGAGATGCTGCTCAATATTTCAACCTCGGTACTTGACGCTAACAG 119

Os_lep_A2 TTATGTACAGACTCTCGGGATGCTGCTCATTATCTCAACTTCGGTACTTAACGCTAACAA 119

Os_lep_C5 TTATGTACAGACTCTCGGGATGCTGCTCATTATCTCAACTTCGGTACTTAACGCTAACAA 119

Os_lep_F4 TTATGTACAGACTCTCGGGATGCTGCTCATTATCTCAACTTCGGTACTTAACGCTAACAA 119

Os_lep_G14 TTATGTACAGACTCTCGGGATGCTGCTCATTATCTCAACTTCGGTACTTAACGCTAACAA 119

Os_lep_C16 TTATGTACAGACTCTCGGGATGCTGCTCATTATCTCAACTTCGGTACTTAACGCTAACAA 119

Os_lep_C7 TTATGTACAGACTCTCGGGATGCTGCTCATTATCTCAACTTCGGTACTTAACGCTAACAA 120

Os_lep_C2 TTATGTACAGACTCTCGGGATGCTGCTCATTATCTCAACTTCGGTACTTAACGCTAACAA 120

Os_lep_C17 TTATGTACAGACTCTCGGGATGCTGCTCATTATCTCAACTTCGGTACTTAACGCTAACAA 120

Os_lep_G9 TTATGTACAGACTCTCGGGATGCTGCTCATTATCTCAACTTCGGTACTTAACGCTAACAA 120

Os_lep_A8 TTATGTACAGACTCTCGGGATGCTGCTCATTATCTCAACTTCGGTACTTAACGCTAACAA 120

Os_lep_A9 TTATGTACAGACTCTCGGGATGCTGCTCATTATCTCAACTTCGGTACTTAACGCTAACAA 120

Os_lep_C8 TTATGTACAGACTCTCGGGATGCTGCTCATTATCTCAACTTCGGTACTTAACGCTAACAA 120

Os_lep_C11 TTATGTACAGACTCTCGGGATGCTGCTCATTATCTCAACTTCGGTACTTAACGCTAACAA 120

Os_lep_F8 TTATGTACAGACTCTCGGGATGCTGCTCATTATCTCAACTTCGGTACTTAACGCTAACAA 119

****** * *** *********** *** ***** ********* *********

Os_lep_F3 TTAAATATGCGTTGTAATTTTGCTTATAGGTCTCCGATACCGTTGTGGARCCCTACAACG 179

Os_lep_A2 TTAAATATGCGATGGAATTTTGCTTATAGGTCTCCGATACCGTTGTGGAGCCCTACAACG 179

Os_lep_C5 TTAAATATGCGATGGAATTTTGCTTATAGGTCTCCGATACCGTTGTGGAGCCCTACAACG 179

Os_lep_F4 TTAAATATGCGATGGAATTTTGCTTATAGGTCTCCGATACCGTTGTGGAGCCCTACAACG 179

Os_lep_G14 TTAAATATGCGATGGAATTTTGCTTATAGGTCTCCGATACCGTTGTGGAGCCCTACAACG 179

Os_lep_C16 TKAAATATGCGATGGAATTTTGCTTATAGGTCTCCGATACCGTTGTGGAGCCCTACAACG 179

Os_lep_C7 TKAAATATGCGATGGAATTTTGCTTATAGGTCTCCGATACCGTTGTGGAGCCCTACAACG 180

Os_lep_C2 TKAAATATGCGATGGAATTTTGCTTATAGGTCTCCGATACCGTTGTGGAGCCCTACAACG 180

Os_lep_C17 TKAAATATGCGATGGAATTTTGCTTATAGGTCTCCGATACCGTTGTGGAGCCCTACAACG 180

Os_lep_G9 TKAAATATGCGATGGAATTTTGCTTATAGGTCTCCGATACCGTTGTGGAGCCCTACAACG 180

Os_lep_A8 TGAAWTATGCGATGGAATTTTGCTTATAGGTCTCCGATACCGTTGTGGAGCCCTACAACG 180

Os_lep_A9 TGAAATATGCGATGGAATTTTGCTTATAGGTCTCCGATACCGTTGTGGAGCCCTACAACG 180

Os_lep_C8 TTAAATATGCGATGGAATTTTGCTTATAGGTCTCCGATACCGTTGTGGAGCCCTACAACG 180

Os_lep_C11 TTAAATATGCGATGGAATTTTGCTTATAGGTCTCCGATACCGTTGTGGAGCCCTACAACG 180

Os_lep_F8 TTAAATATGCGATGGAATTTTGCTTATAGGTCTCCGATACCGTTGTGGAGCCCTACAACG 179

* ** ****** ** ********************************** **********

Os_lep_F3 CCACTCTTTCTGTCCACCAGTTGGTAGAAAATACTGAT**GAG**ACG**TTC** 226

Os_lep_A2 CCACTCTTTCTGTCCACCAGTTGGTAGAAAATACTGAT**GAG**ACG**TTC** 226

Os_lep_C5 CCACTCTTTCTGTCCACCAGTTGGTAGAAAATACTGAT**GAG**ACG**TTC** 226

Os_lep_F4 CCACTCTTTCTGTCCACCAGTTGGTAGAAAATACTGAT**GAG**ACG**TTC** 226

Os_lep_G14 CCACTCTTTCTGTCCACCAGTTGGTAGAAAATACTGAT**GAG**ACG**TTC** 226

Os_lep_C16 CCACTCTTTCTGTCCACCAGTTGGTAGAAAATACTGAT**GAG**ACG**TTC** 226

Os_lep_C7 CCACTCTTTCTGNCCACCAGTTGGTAGAAAATACTGAT**GAG**ACG**TTC** 227

Os_lep_C2 CCACTCTTTCTGTCCACCAGTTGGTAGAAAATACTGAT**GAG**ACG**TTC** 227

Os_lep_C17 CCACTCTTTCTGTCCACCAGTTGGTAGAAAATACTGAT**GAG**ACG**TTC** 227

Os_lep_G9 CCACTCTTTCTGTCCACCAGTTGGTAGAAAATACTGAT**GAG**ACG**TTC** 227

Os_lep_A8 CCACTCTTTCTGTCCACCAGTTGGTAGAAAATACTGAT**GAG**ACG**TTC** 227

Os_lep_A9 CCACTCTTTCTGTCCACCAGTTGGTAGAAAATACTGAT**GAG**ACG**TTC** 227

Os_lep_C8 CCACTCTTTCTGTCCACCAGTTGGTAGAAAATACTGAT**GAG**ACG**TTC** 227

Os_lep_C11 CCACTCTTTCTGTCCACCAGTTGGTAGAAAATACTGAT**GAG**ACG**TTC** 227

Os_lep_F8 CCACTCTTTCTGTCCACCAGTTGGTAGAAAATACTGAT**GAG**ACG**TTC** 226

************ **********************************

***Spiculopteragia asymmetrica Beta-tubulin isoform 1* consensus sequence**

**TTC**TCTGTTGTTCCATCGCCCAAGGTAAATAGCATCTGAAKTTGTCTTGTTTTGTGTCTAAGATAAACAAATGGTCAAGGTAGCTCATGACTTTAGAGTCAAAGAGAGCATAAGTTATTATTATTCAAGTACTCGAAACAGGGTTGTTTAAGGTATCCGACACCGTTGTGGAACCCTACAACGCTACTCTTTCTGTTCACCAATTGGTAGAGAATACCGAT**GAA**ACC**TTC**

***Spiculopteragia asymmetrica* Percent Identity Matrix - created by Clustal2.1**

Sp_asym_E1 100.00 98.25 97.38 99.13 98.25 98.25 98.25 98.25 96.94 98.25 97.38 97.82 97.82 98.25 98.25

Sp_asym_F7 98.25 100.00 96.96 98.26 97.83 97.83 97.83 97.83 96.52 97.83 96.96 97.39 97.39 97.83 97.83

Sp_asym_A 97.38 96.96 100.00 97.39 99.13 99.13 99.13 98.70 97.39 97.83 96.96 97.39 97.39 97.83 97.83

Sp_asym_E2 99.13 98.26 97.39 100.00 98.26 98.26 98.26 98.26 96.96 98.26 97.39 97.83 97.83 98.26 98.26

Sp_asym_D13 98.25 97.83 99.13 98.26 100.00 100.00 100.00 99.57 98.26 98.70 97.83 98.26 98.26 98.70 98.70

Sp_asym_G1 98.25 97.83 99.13 98.26 100.00 100.00 100.00 99.57 98.26 98.70 97.83 98.26 98.26 98.70 98.70

Sp_asym_G2 98.25 97.83 99.13 98.26 100.00 100.00 100.00 99.57 98.26 98.70 97.83 98.26 98.26 98.70 98.70

Sp_asym_G15 98.25 97.83 98.70 98.26 99.57 99.57 99.57 100.00 97.83 99.13 97.39 97.83 97.83 98.26 98.26

Sp_asym_B8 96.94 96.52 97.39 96.96 98.26 98.26 98.26 97.83 100.00 96.96 98.70 97.83 97.83 97.83 97.83

Sp_asym_D14 98.25 97.83 97.83 98.26 98.70 98.70 98.70 99.13 96.96 100.00 98.26 98.70 98.70 99.13 99.13

Sp_asym_B4 97.38 96.96 96.96 97.39 97.83 97.83 97.83 97.39 98.70 98.26 100.00 99.13 99.13 99.13 99.13

Sp_asym_A5 97.82 97.39 97.39 97.83 98.26 98.26 98.26 97.83 97.83 98.70 99.13 100.00 100.00 99.57 99.57

Sp_asym_C3 97.82 97.39 97.39 97.83 98.26 98.26 98.26 97.83 97.83 98.70 99.13 100.00 100.00 99.57 99.57

Sp_asym_A7 98.25 97.83 97.83 98.26 98.70 98.70 98.70 98.26 97.83 99.13 99.13 99.57 99.57 100.00 100.00

Sp_asym_C10 98.25 97.83 97.83 98.26 98.70 98.70 98.70 98.26 97.83 99.13 99.13 99.57 99.57 100.00 100.00

***Spiculopteragia asymmetrica* CLUSTAL O(1.2.4) multiple sequence alignments**

Sp_asym_E1 **TTC**TCTGTTGTTCCATCGCCCAAGGTAAATAGCATCTGAANTTGTCNTGTTTTGTGTCTA 60

Sp_asym_F7 **TTC**TCTGTTGTTCCATCGCCCAAGGTAAATAGCATCTGAAGTTGTCCTGTTKTGTGTCTA 60

Sp_asym_A **TTC**TCTGTTGTTCCATCGCCYAAGGTAAATAGCATCTGAAKTTGTCTTGTTTTGTGTCTA 60

Sp_asym_E2 **TTC**TCTGTTGTTCCATCGCCCAAGGTAAATAGCATCTGAAGTTGTCNTGTTTTGTGTCTA 60

Sp_asym_D13 **TTC**TCTGTTGTTCCATCGCCCAAGGTAAATAGCATCTGAAKTTGTCTTGTTTTGTGTCTA 60

Sp_asym_G1 **TTC**TCTGTTGTTCCATCGCCCAAGGTAAATAGCATCTGAAKTTGTCTTGTTTTGTGTCTA 60

Sp_asym_G2 **TTC**TCTGTTGTTCCATCGCCCAAGGTAAATAGCATCTGAAKTTGTCTTGTTTTGTGTCTA 60

Sp_asym_G15 **TTC**TCTGTTGTTCCATCGCCCAAGGTAAATAGCATCTGAAKTTGTCYTGTTTTGTGTCTA 60

Sp_asym_B8 **TTC**TCTGTTGTTCCATCGCCCAAGGTAAATAGCATCTGAAKTTGTCTTGTTTTGTGTCTA 60

Sp_asym_D14 **TTC**TCTGTTGTTCCATCGCCCAAGGTAAATAGCATCTGAATTTGTCYTGTTTTGTGTCTA 60

Sp_asym_B4 **TTC**TCTGTTGTTCCATCGCCCAAGGTAAATAGCATCTGAATTTGTCTTGTTTTGTGTCTA 60

Sp_asym_A5 **TTC**TCTGTTGTTCCATCGCCCAAGGTAAATAGCATCTGAATTTGTCTTGTTTTGTGTCTA 60

Sp_asym_C3 **TTC**TCTGTTGTTCCATCGCCCAAGGTAAATAGCATCTGAATTTGTCTTGTTTTGTGTCTA 60

Sp_asym_A7 **TTC**TCTGTTGTTCCATCGCCCAAGGTAAATAGCATCTGAATTTGTCTTGTTTTGTGTCTA 60

Sp_asym_C10 **TTC**TCTGTTGTTCCATCGCCCAAGGTAAATAGCATCTGAATTTGTCTTGTTTTGTGTCTA 60

******************** ******************* ***** **** ********

Sp_asym_E1 NGATAAACAAATGGTCAAGGTAGCTCATGACTTTAGAGTCAAAGAGAGCATAAGTT-ATA 119

Sp_asym_F7 GGATAAACAAATGGTCAAGGTAGCTCATGACTTTAGAGTCAAAGAGAGCATAAGTTAATA 120

Sp_asym_A RGATAAACAAATGGTYAAGGTAGCTCATGACTTTAGAGTCAAAGAGAGCATAAGTTAWTA 120

Sp_asym_E2 NGATAAACAAATGGTCAAGGTAGCTCATGACTTTAGAGTCAAAGAGAGCATAAGTTANTA 120

Sp_asym_D13 RGATAAACAAATGGTCAAGGTAGCTCATGACTTTAGAGTCAAAGAGAGCATAAGTTAWTA 120

Sp_asym_G1 RGATAAACAAATGGTCAAGGTAGCTCATGACTTTAGAGTCAAAGAGAGCATAAGTTAWTA 120

Sp_asym_G2 RGATAAACAAATGGTCAAGGTAGCTCATGACTTTAGAGTCAAAGAGAGCATAAGTTAWTA 120

Sp_asym_G15 RGATAAACAAATGGTCAAGGTAGCTCATGACTTTAGAGTCAAAGAGAGCATAAGTTAWTA 120

Sp_asym_B8 RGATAAAMAAATGGTCAAGGTAGCTCATGACTTTAGAGTCAAAGAGAGCATAAGTTATTA 120

Sp_asym_D14 AGATAAACAAATGGTCAAGGTAGCTCATGACTTTAGAGTCAAAGAGAGCATAAGTTAWTA 120

Sp_asym_B4 AGATAAAMAAATGGTCAAGGTAGCTCATGACTTTAGAGTCAAAGAGAGCATAAGTTATTA 120

Sp_asym_A5 AGATAAACAAATGGTCAAGGTAGCTCATGACTTTAGAGTCAAAGAGAGCATAAGTTATTA 120

Sp_asym_C3 AGATAAACAAATGGTCAAGGTAGCTCATGACTTTAGAGTCAAAGAGAGCATAAGTTATTA 120

Sp_asym_A7 AGATAAACAAATGGTCAAGGTAGCTCATGACTTTAGAGTCAAAGAGAGCATAAGTTATTA 120

Sp_asym_C10 AGATAAACAAATGGTCAAGGTAGCTCATGACTTTAGAGTCAAAGAGAGCATAAGTTATTA 120

****** ******* **************************************** **

Sp_asym_E1 TTATTCAAGTACTCGAAACAGGGTTGTTTAAGGTATCCGACACCGTTGTGGAACCCTACA 179

Sp_asym_F7 TTATTCAAGTACTCGAAACAGGGTTGTTTAAGGTATCCGACACCGTTGTGGAACCCTACA 180

Sp_asym_A TTATTCAAGTACTCGAAACAGGGTTGTTTAAGGTATCCGACACCGTTGTGGAACCCTACA 180

Sp_asym_E2 TTATTCAAGTACTCGAAACAGGGTTGTTTAAGGTATCCGACACCGTTGTGGAACCCTACA 180

Sp_asym_D13 TTATTCAAGTACTCGAAACAGGGTTGTTTAAGGTATCCGACACCGTTGTGGAACCCTACA 180

Sp_asym_G1 TTATTCAAGTACTCGAAACAGGGTTGTTTAAGGTATCCGACACCGTTGTGGAACCCTACA 180

Sp_asym_G2 TTATTCAAGTACTCGAAACAGGGTTGTTTAAGGTATCCGACACCGTTGTGGAACCCTACA 180

Sp_asym_G15 TTATTCAAGTACTCGAAACAGGGTTGTTTAAGGTATCCGACACCGTTGTGGAACCCTACA 180

Sp_asym_B8 TTATTCAAGTACTCRAAACAGGGTTGTTTAAGGTATCCGACACCGTTGTGGAACCMTACA 180

Sp_asym_D14 TTATTCAAGTACTCGAAACAGGGTTGTTTAAGGTATCCGACACCGTTGTGGAACCCTACA 180

Sp_asym_B4 TTATTCAAGTACTCGAAACAGGGTTGTTTAAGGTATCCGACACCGTTGTGGAACCMTACA 180

Sp_asym_A5 TTATTCAAGTACTCGAAACAGGGTTGTTTAAGGTATCCGACACCGTTGTGGAACCNTACA 180

Sp_asym_C3 TTATTCAAGTACTCGAAACAGGGTTGTTTAAGGTATCCGACACCGTTGTGGAACCNTACA 180

Sp_asym_A7 TTATTCAAGTACTCGAAACAGGGTTGTTTAAGGTATCCGACACCGTTGTGGAACCCTACA 180

Sp_asym_C10 TTATTCAAGTACTCGAAACAGGGTTGTTTAAGGTATCCGACACCGTTGTGGAACCCTACA 180

************** **************************************** ****

Sp_asym_E1 ACGCTACTCTTTCTGTTCACCAATTGGTAGAGAATACCGAT**GAA**ACC**TTC** 229

Sp_asym_F7 ACGCTACTCTTTCTGTTCACCAATTGGTAGAGAATACCGAT**GAA**ACC**TTC** 230

Sp_asym_A ACGCTACTCTTTCTGTTCACCAATTGGTAGAGAATACCGAT**GAA**ACC**TTC** 230

Sp_asym_E2 ACGCTACTCTTTCTGTTCACCAATTGGTAGAGAATACCGAT**GAA**ACC**TTC** 230

Sp_asym_D13 ACGCTACTCTTTCTGTTCACCAATTGGTAGAGAATACCGAT**GAA**ACC**TTC** 230

Sp_asym_G1 ACGCTACTCTTTCTGTTCACCAATTGGTAGAGAATACCGAT**GAA**ACC**TTC** 230

Sp_asym_G2 ACGCTACTCTTTCTGTTCACCAATTGGTAGAGAATACCGAT**GAA**ACC**TTC** 230

Sp_asym_G15 ACGCTACTCTTTCTGTTCACCAATTGGTAGAGAATACCGAT**GAA**ACC**TTC** 230

Sp_asym_B8 ACGCTACTCTTTCTGTTCACCAATTGGTAGAGAATACCGAT**GAA**ACC**TTC** 230

Sp_asym_D14 ACGCTACTCTTTCTGTTCACCAATTGGTAGAGAATACCGAT**GAA**ACC**TTC** 230

Sp_asym_B4 ACGCTACTCTTTCTGTTCACCAATTGGTAGAGAATACCGAT**GAA**ACC**TTC** 230

Sp_asym_A5 ACGCTACTCTTTCTGTTCACCAATTGGTAGAGAATACCGAT**GAA**ACC**TTC** 230

Sp_asym_C3 ACGCTACTCTTTCTGTTCACCAATTGGTAGAGAATACCGAT**GAA**ACC**TTC** 230

Sp_asym_A7 ACGCTACTCTTTCTGTTCACCAATTGGTAGAGAATACCGAT**GAA**ACC**TTC** 230

Sp_asym_C10 ACGCTACTCTTTCTGTTCACCAATTGGTAGAGAATACCGAT**GAA**ACC**TTC** 230

**************************************************
